# Effect of gillnet mesh size on the capture probability and capture patterns in the Asian paddle crab (*Charybdis japonica*) fishery

**DOI:** 10.1016/j.heliyon.2024.e25771

**Published:** 2024-02-04

**Authors:** Mengjie Yu, Bent Herrmann, Kristine Cerbule, Changdong Liu, Liyou Zhang, Yanli Tang

**Affiliations:** a Fisheries College, Ocean University of China, 266003, Qingdao, Shandong, China; b SINTEF Ocean, Fishing Gear Technology, Willemoesvej 2, 9850, Hirtshals, Denmark; c UiT the Arctic University of Norway, Breivika, N-9037, Tromsø, Norway; d DTU Aqua, Technical University of Denmark, Hirtshals, Denmark

**Keywords:** Gillnets, Mesh size, Catch efficiency, Capture patterns, *Charybdis japonica*

## Abstract

In the Asian paddle crab (*Charybdis japonica*) gillnet fishery in the Yellow Sea, China, the minimum mesh size (MMS) regulation has been of a major importance due to high bycatch rates of undersized crabs. In this study, we evaluated how gillnet mesh size can affect the capture probability of *C. japonica* and capture patterns in this fishery by comparing the performance of gillnets with four different mesh sizes (60, 70, 80, and 90 mm). Our results showed that changes in gillnet mesh size significantly affect the capture probability of different sizes of crabs. Specifically, increased mesh size decreased the capture probability of undersized crabs and their fraction in the catches decreased from 64 % to 24 % when mesh size was increased from 60 mm to 90 mm. In contrast, gillnets with larger mesh sizes significantly improved the capture probability of legal-sized crabs. Moreover, no significant differences were observed for the species catch composition between gillnets of different mesh sizes. Based on these results, we recommend 90 mm as the MMS in gillnets to improve sustainability in *C. japonica* fishery.

## 1 Introduction

The Asian paddle crab (*Charybdis japonica*) is a benthic crustacean species, belonging to the family *Portunidae*. *C. japonica* is native to the coast of Japan and has extended its distribution range to Korea, Southeast Asia, Oceania, and the Yellow Sea, Bohai Sea, and East Sea of China. This species inhabits a large variety of habitats, including sandy, muddy, and rocky bottoms [[1], [2], [3], [4], [5], [6], [7], [8]]. In coastal China, *C. japonica* is an important commercial species because of its high nutritional and economic value. Therefore, it constitutes an essential part of crustacean fisheries in this area [9]. The annual total landings of *C. japonica* have been fluctuating between 2.2 × 10^4^ t and 3.5 × 10^4^ t in the period between 2017 and 2022 [10]. Nowadays, with the dramatic decline in biomass of many commercially important fish species, *C. japonica* and other crustacean species have a considerable importance in providing the seafood security [11,12]. Almost all production of *C. japonica* is taking place as marine capture fisheries, as the aquaculture sector for this species is limited. This fishery targets *C. japonica* using different types of fishing gear, including stow nets, traps, pots, trammel nets, and gillnets.

Generally, gillnets are considered a low cost, easy operation fishing gear [13,14]. Therefore, bottom-set gillnets are one of the main fishing gears used for targeting *C. japonica* in the coastal waters of the Yellow Sea, China [15]. In this fishery, the Chinese Ministry of Agriculture has implemented a minimum landing size (MLS) of 50 mm CL since 2004 [7]. In addition, in 2014, Shandong province issued a local regulation stating that the bycatch ratio of undersized crabs should not exceed 25 % of the total crab catches [16]. However, currently there is no minimum mesh size (MMS) regulation specified for this fishery to supplement these MLS and bycatch ratio regulations.

Therefore, in this fishery, the fishers tend to choose gillnets with different mesh sizes. The mesh sizes usually range from 60 to 90 mm, and sometimes a combination of gillnet sheets of different mesh sizes is used aiming to maximize the catches. However, since the optimal gillnet mesh size for this fishery is not scientifically established, the current use of different mesh-sized gillnets often results in severe bycatch and discard issues of undersized crabs. Undersized crabs are often damaged when removed from gillnets and returned to the sea. These injuries can cause mortality or delayed mortality after being released at sea or can affect somatic growth, which in turn can have a negative effect on the yield from the fishery. Furthermore, disentangling and sorting out juvenile crabs from the gillnets when the gear is retrieved is a time- and labor-intensive process onboard the fishing vessels which is prone to damage the nets. Additionally, the market prices of different-sized crabs vary greatly. According to the seafood market survey in Shandong province, legal-sized individuals at *CL* 50–65 mm can be sold at 5 yuan/ind, while for larger individuals (*CL* > 65 mm), the price can be doubled (i.e., 10 yuan/ind). In comparison, undersized crabs (*CL* < 50 mm) will have a price that is below 1 yuan/ind. Therefore, fishers prefer to capture crabs of larger *CL* sizes and further have interest to improve the capture rates of legal-sized individuals.

The capture mechanisms in gillnets can vary greatly for different species based on their morphological and behavioral characteristics. For fish species, the individuals are often caught in gillnets by gilling, wedging, entangling, and snagging depending on how the fish are enmeshed in the gillnet netting [17,18]. For instance, for some roundfish species, gilling is the most dominant way for capture [19,20]. Therefore, a specific gillnet mesh size corresponds to a certain fish length that gets caught most effectively. Thus, the gillnet mesh size is an important variable determining the catch efficiency and selectivity of the gear. Compared to fish, crabs can be more easily caught by entangling in gillnets due to their non-compressible exoskeleton and multiple limbs, claws and spines. Increased entanglements may negatively affect the gillnet selectivity for crabs. However, previous studies showed that increasing mesh sizes could improve the gillnet selectivity for crabs [14,21]. This indicates that there can be differences in length-dependent capture probability of crabs between gillnets of different mesh sizes, including in the *C. japonica* gillnet fishery.

Since this gillnet fishery commonly captures several species, the effect of changing the gillnet mesh size must be investigated on the entire catch composition rather than focusing only on the primary target species. Specifically, *C. japonica* gillnet fishery also captures a wide range of bottom-dwelling finfish and echinoderm species, and other marine organisms inhabiting similar areas as *C. japonica*. Currently, there are no regulations regarding the capture of bycatch species in this fishery. Some of the common bycatch species are of commercial value and thus landed by fishers for sale or consumption (e.g., *Sebastes schlegelii* and *Hexagrammos otakii*). However, the unwanted bycatch species are often discarded by fishers. Additionally, the bycatch of echinoderms (e.g., *Mesocentrotus nudus*) can cause operational challenges for fishers because they are difficult to disentangle from gillnets, thus easily damaging the nets. Since the catch composition can potentially vary between gillnets of different mesh sizes [22,23], this aspect needs to be considered when evaluating the effect of gillnet mesh size. This can have implications for maintaining additional benefits for fishers, protecting biodiversity, and formulating bycatch management regulations.

In this study, we conducted the first scientific investigation on the capture probability and capture patterns of gillnets with different mesh sizes in the *C. japonica* gillnet fishery in the Yellow Sea, China. We tested and compared the catch performance of gillnets with mesh sizes commonly used in this fishery (60, 70, 80, and 90 mm). Therefore, our study was designed to answer the following questions:

• Are there any differences in the length-dependent capture probability of *C. japonica* between gillnets of different mesh sizes?
• Are the capture patterns affected by the gillnet mesh size?
• What is the optimal mesh size for the *C. japonica* gillnet fishery among the mesh sizes that are commonly used in the commercial fishery?

## 2 Materials and methods

### 2.1 Sea trials

**Fig. 1:**
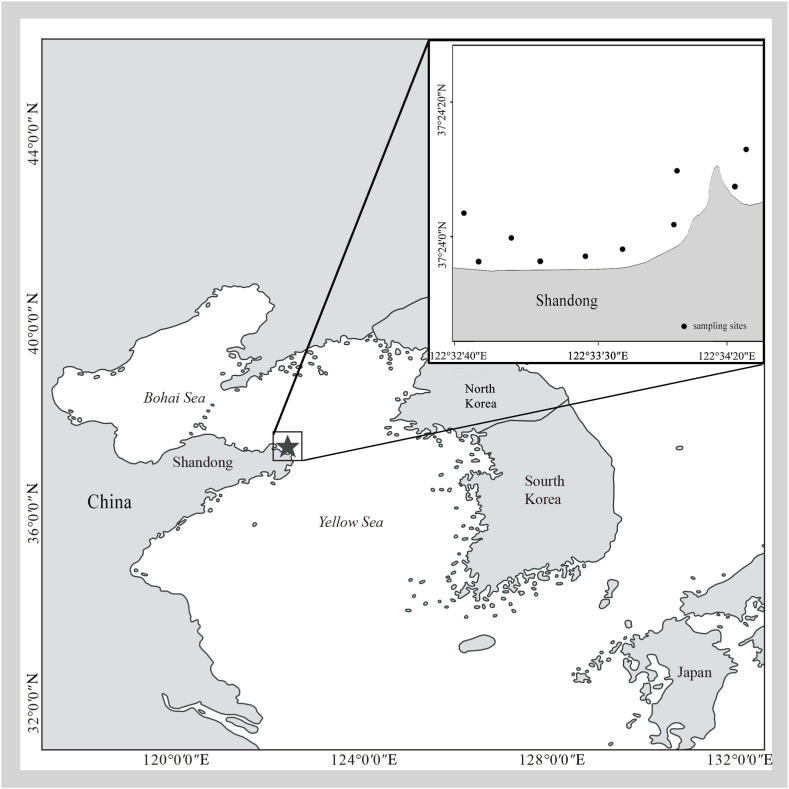
Map of study area in the Yellow Sea of China where the gillnets were deployed.

Sea trials were conducted in October 2020 in the coastal waters of the Yellow Sea, China (122°32′40″-122°34′40″E, 37°23′40″-37°24′20″N) (Fig. 1). The study area is an important commercial fishing ground for targeting *C. japonica*. The substrate consist of a mixture of mud, sand, and rock. The water depth varies from 5 to 20 m. A commercial fishing vessel, “Lurongyuyang 62705” (LOA 6.7 m, width 2.2 m, height 0.8 m, weight 2.0 GT, power 5.8 kW), was used to deploy and retrieve the gillnets during the sea trials.

**Fig. 2:**
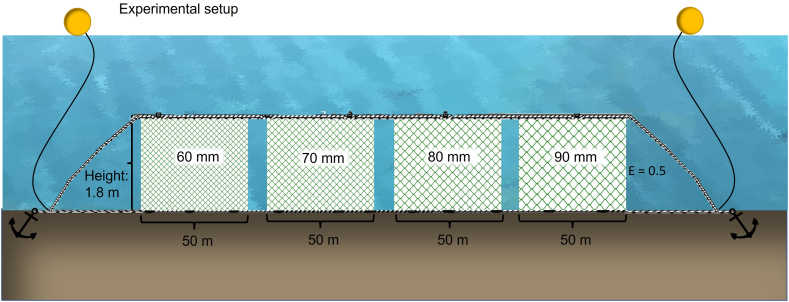
Experimental setup showing gillnets with different mesh sizes (60, 70, 80 and 90 mm mesh size) deployed as a fleet during the fishing trials. Three such fleets were deployed for each deployment in these trials simultaneously and in the same fishing area. E = hanging ratio.

Gillnets with 60, 70, 80 and 90 mm fully stretched mesh size were used in these trials (herein M60, M70, M80, and M90, respectively). All gillnets were made of green nylon monofilament with twine thickness ranging from 0.20 to 0.23 mm (Table 1). In total, 12 gillnet sheets were used in this study, with three replicates of each of the four mesh sizes. All gillnets were divided into three fleets, each containing one replicate for each mesh size. Therefore, each fleet consisted of four gillnet sheets arranged in the following order: M60, M70, M80, and M90 (Fig. 2). Each gillnet sheet measured 50 m in length and 1.8 m in height with a hanging ratio (E) of 0.50 (Fig. 2). The float, constructed from plastic foam, provided a buoyancy of 80 g/m, and the sinker consisted of 500 lead blocks, each weighing 20 g. The specifications and design parameters of the tested gillnets were identical to those of the commercial gillnets, including material, twine thickness, twine color, hanging ratio, float-sink ratio, and dimensions (Table 1). Two buoys and anchors each weighing 15 kg were connected to each end of the fleet (Fig. 2).

**Table 1:** Specifications of the tested gillnets with 60 mm (M60), 70 mm (M70), 80 mm (M80) and 90 mm (M90) mesh size. SE represents standard errors.

	Mesh size ± SE (mm)	Twine thickness ± SE (mm)	Mesh number in length	Mesh number in height
M60	58.92 ± 0.67	0.20 ± 0.09	1697	35
M70	71.22 ± 0.89	0.21 ± 0.08	1404	29
M80	80.07 ± 0.53	0.21 ± 0.09	1249	26
M90	89.91 ± 0.31	0.23 ± 0.06	1112	23

In alignment with the standard commercial fishing procedures, gillnets were deployed at twilight and retrieved the following morning, allowing for an approximate soak time of 12 h. Before each deployment, we inspected all nets and replaced the broken ones. After each trial, all *C. japonica* were sorted by gillnet mesh sizes and measured for *CL* to the nearest mm using calipers. All bycatch species were identified, counted, and documented.

### 2.2 Modeling the length-dependent capture probability

In this study we estimated, conditioned capture, the length-dependent capture probability in gillnets of different mesh size [19]. Each deployment for all gillnet fleets in each fishing day were considered as the base unit for the analysis. The analysis was conducted independently for each mesh size following the description below [19]. Specifically, conditioned capture, the expected capture probability (*CP*) for the mesh size *m* for crab with carapace length *cl* will be [19]:

(1) $$\begin{array}{c} {CP}_{m,cl}=\frac{\sum_{j=1}^{h}n_{m,cl,j}}{\sum_{j=1}^{h}\sum_{i=1}^{M}n_{i,cl,j}} \end{array}$$

where $n_{m,cl,j}$ is the number *n* of crab captured in the gillnet with mesh size *m* belonging to the carapace length class *cl* in gillnet deployment *j*. In Equation (1), *M* is the total number of tested gillnet mesh sizes, and *h* is the total number of deployments. The functional form of the capture probability ${CP}_{m}\left(cl,w\right)$ was obtained using the maximum likelihood estimation by minimizing the following expression:

(2) $$\begin{array}{c} -\sum_{j=1}^{h}\sum_{cl}\left\{n_{m,cl,j}\times \ln\;\left[{CP}_{m}\left(cl,w\right)\right]+\left[-n_{m,cl,j}+\sum_{i=1}^{M}n_{i,cl,j}\right]\times \ln\left[1.0-{CP}_{m}\left(cl,w\right)\right]\right\} \end{array}$$

where ***w*** represents the parameters that describe the capture probability curve defined by ${CP}_{m}\left(cl,w\right)$. Due to the applied experimental design using gillnets with four different mesh sizes, the expected value for equal capture probability between the gillnets would be 0.25. Specifically, if the gillnets with one of the mesh sizes for some *C. japonica* length classes catches more than the average for the four compared mesh size nets, then ${CP}_{m}\left(cl,w\right)$ would be significantly larger than 0.25. In contrast, a ${CP}_{m}\left(cl,w\right)$ value significantly lower than 0.25 would show that the specific gillnet type captures significantly less *C. japonica* compared the other gillnets [20].

Equation (1) and Expression (2) together have a similar form to what is commonly used for modeling and estimating the length-dependent catch comparison rate between two fishing gears [24]. Thus, we applied the same technique to model ${CP}_{m}\left(cl,w\right)$ as is often used in catch comparison studies based on binominal count data [25] by using:

(3) $$\begin{array}{c} {CP}_{m}\left(cl,w\right)=\frac{\exp\left[f\left(cl,w_{0},\ldots ,w_{q}\right)\right]}{1+\exp\left[f\left(cl,w_{0},\ldots ,w_{q}\right)\right]} \end{array}$$

In Equation (3), *f* is a polynomial of order *q* with coefficients from $w_{0}$ to $w_{q}$. The values of the parameters ***w*** describing ${CP}_{m}\left(cl,w\right)$ were estimated by minimizing Expression (2). We considered *f* of up to an order of 4 with parameters $w_{0}$-$w_{4}$. Leaving out one or more of the parameters $w_{0}\ldots w_{4}$ resulted in 31 additional candidate models for the capture probability ${CP}_{m}\left(cl,w\right)$. Estimations of capture probability were made using multi-model inference to obtain the optimal combined model [25,26]. The ability of the combined model to describe the experimental data was evaluated based on the *p*-value. The *p*-value, which can be calculated based on the model deviance and the degrees of freedom [25,27], should be larger than 0.05 for the combined model to fit the data well. A double bootstrap method (1000 bootstrap repetitions) was used to estimate the Efron percentile 95 % confidence intervals (CIs) [28] for the length-dependent capture probability curves by incorporating both within- and between-deployments variations [[29], [30], [31]].

Further, the pairwise differences (delta) in length-dependent capture probability between gillnets with different mesh sizes ΔCP(cl) were estimated as follows:

(4) $$\begin{array}{c} \Delta CP\left(cl\right)={CP}_{B}\left(cl\right)-{CP}_{A}\left(cl\right) \end{array}$$

In Equation (4), ${CP}_{A}\left(cl\right)$ is the capture probability for gillnet with mesh size *A* (60, 70, 80 or 90 mm), and ${CP}_{B}\left(cl\right)$ represents the capture probability for gillnet *B* with a different mesh size (60, 70, 80 or 90 mm mesh size, respectively). The Efron percentile 95 % CIs for the ΔCP(cl) were estimated based on the two bootstrap population results for ${CP}_{A}\left(cl\right)$ and ${CP}_{B}\left(cl\right)$. Since they were acquired independently, it is valid to create a new bootstrap population of results for ΔCP(cl) [32,33]. Significant differences between the different mesh size gillnets were obtained if the 95 % CIs for delta values did not include 0.0.

### 2.3 Estimation of exploitation pattern indicators

To investigate how gillnet mesh size would affect the exploitation patterns in the *C. japonica* fishery, we estimated the value of three exploitation pattern indicators, *nP−*, *nP+* and *nDRatio*. Specifically, to quantify the effect of gillnet mesh size for catches of *C. japonica*, conditioned capture by one of the gillnet mesh size *m*, we estimated the exploitation pattern indicators quantifying the average percentage of undersized (${NP-\;}_{m}$) and legal-sized (${NP+}_{m}$) individuals captured with that specific mesh size, and the discard ratio $({ndRatio}_{m}$). The discard ratio is quantifying the fraction of undersized *C. japonica* in the catch (in %) caught within the specific mesh size gillnet. The three exploitation pattern indicators (${NP-\;}_{m}$, ${NP+}_{m}$, and ${ndRatio}_{m}$) were estimated as follows:

$${NP-\;}_{m}=100\times \frac{\sum_{j}\sum_{Cl<MLS}n_{m,cl,j}}{\sum_{j}\sum_{Cl<MLS}\sum_{i=1}^{M}n_{i,cl,j}}$$

(5) $$\begin{array}{c} {NP+}_{m}=100\times \frac{\sum_{j}\sum_{Cl\geq MLS}n_{m,cl,j}}{\sum_{j}\sum_{Cl\geq MLS}\sum_{i=1}^{M}n_{i,cl,j}} \end{array}$$

$${ndRatio}_{m}=100\times \frac{\sum_{j}\sum_{Cl<MLS}n_{m,cl,j}}{\sum_{j}\sum_{Cl}\sum_{i=1}^{M}n_{i,cl,j}}$$

Ideally *nP−*_*m*_ and *nDRatio*_*m*_ should be low (close to 0), while *nP* + _*m*_ should be as high as possible (i.e., close to 100) [34]. The Efron percentile 95 % CIs of these indicators in Equation (5) were estimated using the double bootstrap method as described above. However, these indicators are specific to the size structure of the crab population present in the fishing grounds. Therefore, they provide an estimate that is specific for the targeted population and that cannot be extrapolated to other fishing areas and seasons [35,36].

### 2.4 Length frequency distributions

Further, to calculate the proportion of the total catch of *C. japonica* for and up to a given carapace length class *cl*, conditioned capture in gillnet with mesh size *m*, the length frequency distribution and cumulative length frequency distribution analysis was performed using the following equation:

(6) $$\begin{array}{c} {Dn}_{m,cl}=\frac{\sum_{j=1}^{h}n_{m,cl,j}}{\sum_{j=1}^{h}\sum_{cl}n_{m,cl,j}}\\ {CDn}_{m,CL}=\frac{\sum_{j=1}^{h}\sum_{cl=0}^{CL}n_{m,cl,j}}{\sum_{j=1}^{h}\sum_{cl}n_{m,cl,j}} \end{array}$$

The 95 % CIs for the ${Dn}_{m,cl}$ and ${CDn}_{m,CL}$ can be obtained using the double bootstrap method by integrating the evaluation of Equation (6). Further, we quantified the differences in length frequency $\Delta {Dn}_{m,cl}$ and cumulative length frequency $\Delta {CDn}_{m,CL}$ between gillnets with different mesh sizes *A* and *B* using the following equation:

$$\begin{array}{c} {\Delta Dn}_{A,B,cl}={Dn}_{B,cl}-{Dn}_{A,cl} \end{array}$$

(7) $$\begin{array}{c} {\Delta CDn}_{A,B,CL}={CDn}_{B,CL}-{CDn}_{A,CL} \end{array}$$

We used the double bootstrap method mentioned above to estimate the Efron 95 % CIs for the ${\Delta Dn}_{A,B,cl}$ and ${\Delta CDn}_{A,B,CL}$ in Equation (7).

### 2.5 Species dominance analysis

Finally, we estimated species compositions in gillnets with different mesh sizes to examine potential differences in species dominance patterns. Cumulative dominance analysis is a standard method for quantifying relative abundance of species in samples, and it has been commonly used for comparing fishing gear catches [37,38]. In this study, we utilized cumulative dominance curves, plotting the cumulative proportional abundances against a fixed species rank, based on the number of each species caught in gillnets of different mesh sizes. This approach provides an overview on how many species are dominant and their relative dominance distribution in the catches of gillnets with different mesh sizes. Detailed information regarding the procedure for species dominance analysis is elaborated in published literatures [[37], [38], [39], [40]].

All the data analysis procedures (Sections 2.2-2.5) were conducted using the statistical software SELNET [29].

## 3 Results

### 3.1 Description of sea trials and catches

During the sea trials, a total of ten valid hauls were carried out. The water depth varied from 10.5 to 13.7 m, and the soak time for the gillnets ranged between 11.7 and 12.4 h (Table 2). In total, 1771 *C. japonica* were captured in all gillnets with *CL* size ranging from 28 to 75 mm (Table 2). In this study, we did not discriminate between male and female crabs since the relevant MLS regulation for *C. japonica* in this fishery is specified only for the crab *CL* size. Additionally, throughout the experiments, seven bycatch species were recorded (Table 3).

**Table 2:** Summary details of the catch data of *C. japonica* in the sea trials.

Trip ID	Date	Soak time (h)	Depth (m)	Total number of *C. japonica* caught			
				M60	M70	M80	M90
1	October 21, 2020	12.1	11.3	43	46	40	45
2	October 22, 2020	12.0	12.2	43	47	44	53
3	October 23, 2020	11.8	10.9	38	42	43	51
4	October 24, 2020	12.2	13.7	34	44	43	41
5	October 25, 2020	12.4	12.9	42	49	37	45
6	October 26, 2020	12.3	10.5	33	50	39	46
7	October 27, 2020	11.7	11.8	43	45	38	42
8	October 28, 2020	12.0	13.2	38	47	44	41
9	October 29, 2020	11.9	11.1	43	44	40	46
10	October 30, 2020	12.1	12.8	51	44	74	53
Total				408	458	442	463

**Table 3:** List of bycatch species and number of individuals captured for the four mesh sizes during the experiments. Species names marked with * denote species of wanted catch.

Species name	Common name	Number of individuals			
		M60	M70	M80	M90
*Sebastes schlegelii* (Hilgendorf, 1880) *	Black rockfish	13	9	17	15
*Hexagrammos otakii* (Jordan & Starks, 1895) *	Fat greenling	9	14	10	11
*Mesocentrotus nudus* (A. Agassiz, 1864)	Sea urchin	3	0	3	0
*Hexagrammos agrammus* (Temminck & Schlegel, 1843)	Spotty belly greenling	14	10	16	13
*Pseudopleuronectes yokohamae* (Günther, 1877)	Marbled flounder	0	2	5	3
*Patiria pectinifera* (Muller & Troschel, 1842)	Starfish	2	0	1	1
*Platichthys bicoloratus* (Basilewsky, 1855)	Stone flounder	1	1	0	2

### 3.2 Length-dependent capture probability

**Fig. 3:**
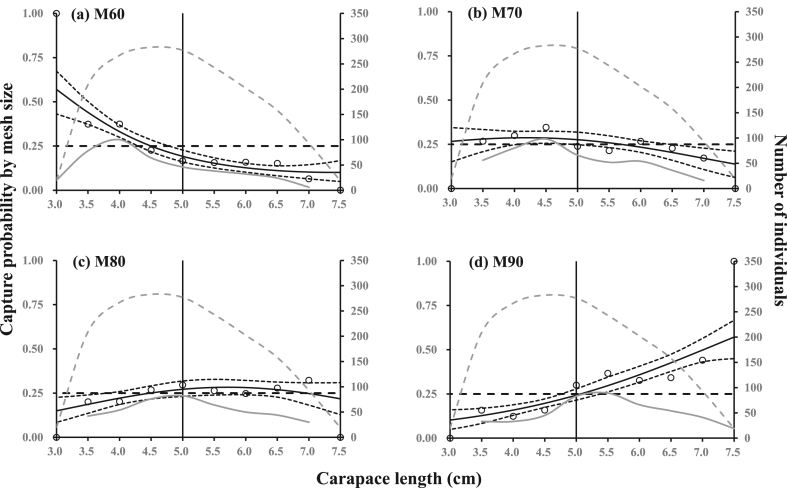
Length-dependent capture probability of the four different gillnet mesh sizes, (a) 60 mm, (b) 70 mm, (c) 80 mm and (d) 90 mm, for Asian paddle crab (*Charybdis japonica*). Circles represent experimental rates. Thick solid curves represent the modeled length-dependent capture probability. Dashed curves represent 95 % confidence intervals. Gray dashed lines represent summed captured population of the four mesh sizes. Gray solid lines represent the population of the specific mesh size. Vertical solid lines represent the minimum landing size (MLS) of Asian paddle crab. Horizontal dashed lines indicate equal catch efficiency of four mesh sizes.

For all mesh sizes, the estimated *p*-value was below 0.05 (Table 4). However, the capture probability curve represented the trends in experimental data well (Fig. 3); therefore, the low *p*-value was assumed to be due to overdispersion in the data [27].

**Table 4:** Fit statistics of the length-dependent capture probability analysis and estimated exploitation pattern indicators for the four mesh sizes. DOF denotes degrees of freedom.

	M60	M70	M80	M90
*p*-value	<0.0001	<0.0001	0.0005	<0.0001
Deviance	40.25	28.31	21.89	49.22
DOF	5	5	5	5
*NP-* (%)	33.63 (30.20–37.28)	30.04 (25.98–34.28)	22.08 (18.62–25.62)	14.25 (11.13–17.38)
*NP+* (%)	14.72 (12.22–17.27)	22.58 (19.91–25.71)	27.22 (23.50–31.14)	35.48 (32.04–39.25)
*ndRatio* (%)	64.22 (58.91–69.53)	51.09 (45.90–55.60)	38.91 (34.41–43.84)	23.97 (19.26–28.51)

The results of this study showed that M60 had a significantly higher capture probability for undersized crabs and a lower capture probability for legal-sized individuals compared to gillnets with larger mesh sizes (Fig. 3). In contrast, for M90, the capture probability of undersized crabs was significantly lower, and it exhibited an increasing trend with the increment of length classes. M70 and M80 did not show an obvious length-dependent capture probability. Specifically, for almost all length classes of *C. japonica* captured by M70 and M80 gillnets, the 95 % CIs included the baseline for equal capture probability (0.25). Therefore, this showed that there were no significant differences in capture probability by M70 and M80 gillnets except for a small range of *C. japonica* length classes (i.e., 65–75 mm for M70 and 30–35 mm for M80).

**Fig. 4:**
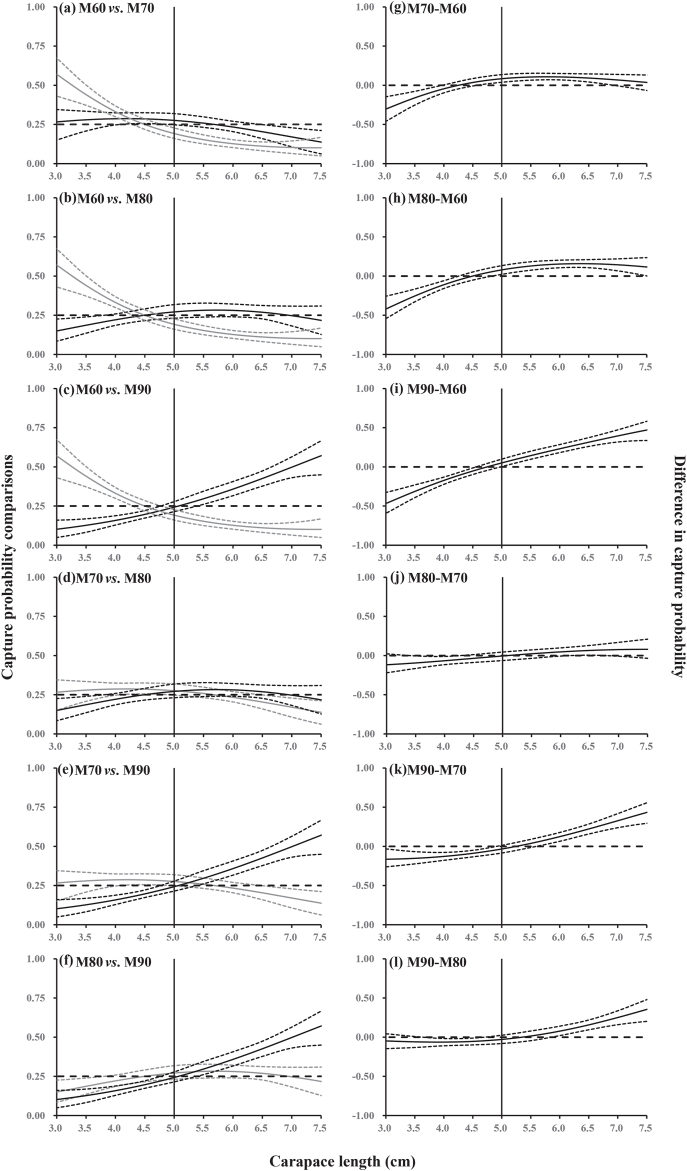
Capture probability comparisons between the four mesh sizes. Left panel (a–f): the curves (solid lines) with 95 % confidence intervals (dotted lines) represent the modeled capture probability for the smaller gillnet mesh size (gray), and the larger mesh size (black). Right panel (g–l): the curves (solid lines) with 95 % confidence intervals (dotted lines) represent the differences in capture probability between different gillnet mesh sizes. Vertical solid lines represent the minimum landing size of Asian paddle crab (*Charybdis japonica*). Horizontal dashed lines represent the baseline at which the gillnets with different mesh size have equal capture probability.

The pairwise differences (delta) in length-dependent capture probability between the four gillnet mesh sizes are shown in Fig. 4. The results showed that gillnets with the smallest mesh size (M60) captured a larger number of undersized crabs compared to the other gillnets. Meanwhile, M70, M80, and M90 had significantly higher capture probability for legal-sized crabs than M60 at the following length classes: 50–65 mm for M70, 50–75 mm for M80, and 50–75 mm for M90. In the comparison between M70 and M80 gillnets, significant differences in capture probability were only observed for *C. japonica* length classes between 35 and 40 mm. As for M90 *vs*. M70 and M90 *vs*. M80, the largest mesh size gillnets would have significantly lower capture probability for undersized crabs (for *CL* between 30-50 mm and 40–45 mm) compared to M70 and M80, respectively. Simultaneously, the largest mesh size gillnets had higher capture probability for crabs at length classes between 60 and 75 mm than both, M70 and M80 gillnets (Fig. 4).

### 3.3 Exploitation pattern indicators

The exploitation pattern indicators showed that using larger mesh sizes would significantly reduce the capture probability of undersized crabs, as showed by the lower values of exploitation pattern indicators quantifying the average percentage of undersized individuals (*NP*-_*m*_) (Table 4). For instance, the M60 gillnets captured 33.63 % (CI: 30.20–37.28 %) of the total undersized crabs, while the value of *NP*-_*m*_ was significantly lower for the M90 gillnets (i.e., 14.25 % (CI: 11.13–17.38 %); Table 4). The opposite was observed for legal-sized crabs where gillnets with larger mesh sizes captured higher proportions of legal-sized *C. japonica* as reflected by (*NP* + _*m*_) (Table 4). *ndRatio*_*m*_ showed that the proportion of undersized crabs caught within each mesh size significantly decreased with increasing mesh sizes used in these trials. Specifically, *ndRatio*_*m*_ decreased from 64.22 % (CI: 58.91–69.53 %) in M60 to 23.97 % (CI: 19.26–28.51 %) in M90 gillnets (Table 4).

### 3.4 Length frequency distributions

**Fig. 5:**
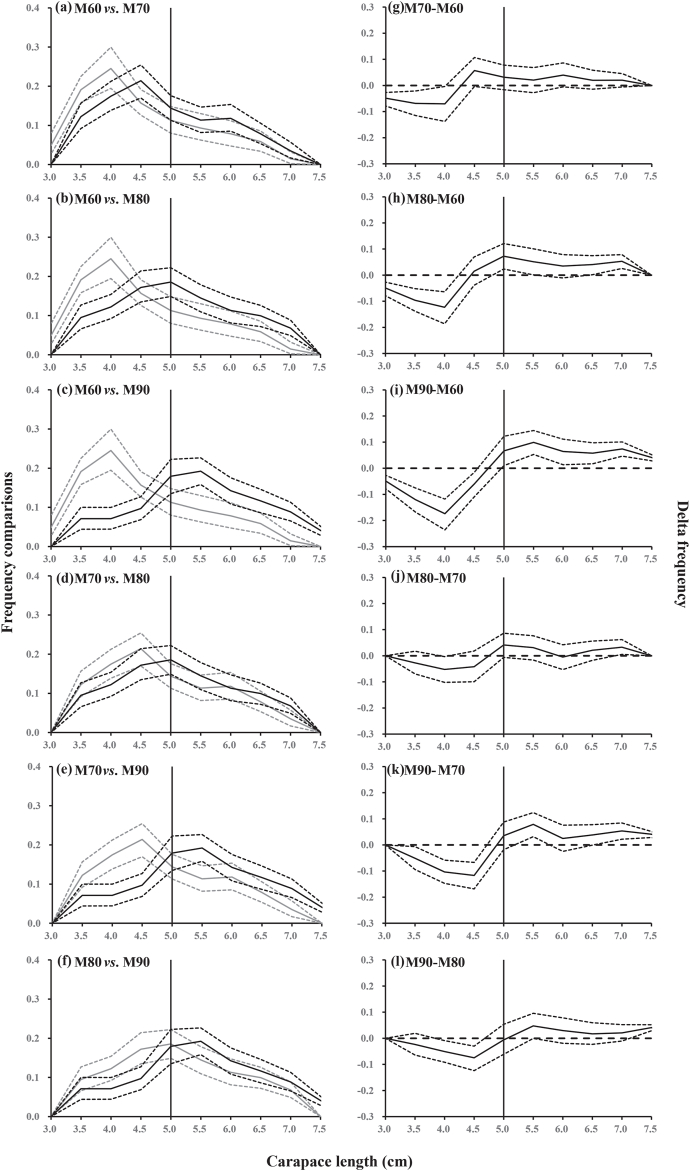
Length frequency distributions between the four gillnet mesh sizes (60, 70, 80 and 90 mm). Left panel (a–f): length frequency distribution curves (solid lines) with 95 % confidence intervals (dotted lines) representing the estimated length frequency for the smaller gillnet mesh size (gray), and the larger mesh size (black). Right panel (g–l): length frequency distribution curves (solid lines) with 95 % confidence intervals (dotted lines) represent the differences in length frequency between different gillnet mesh sizes. Vertical solid lines represent the minimum landing size of Asian paddle crab (*Charybdis japonica*). Horizontal dashed lines are baseline for no difference in length frequency distribution between the two mesh sizes.

The length distributions of *C. japonica* captured in gillnets with different mesh sizes were significantly different (Fig. 5). Specifically, the modal length class (the size class being the most frequent in the data set) of *C. japonica* gradually increased with increasing gillnet mesh sizes. In M60 and M70 gillnets, the modal length of *C. japonica* was far below the MLS. The modal length was equal to the MLS in M80 gillnet. However, in M90 gillnet, the modal length exceeded the MLS.

**Fig. 6:**
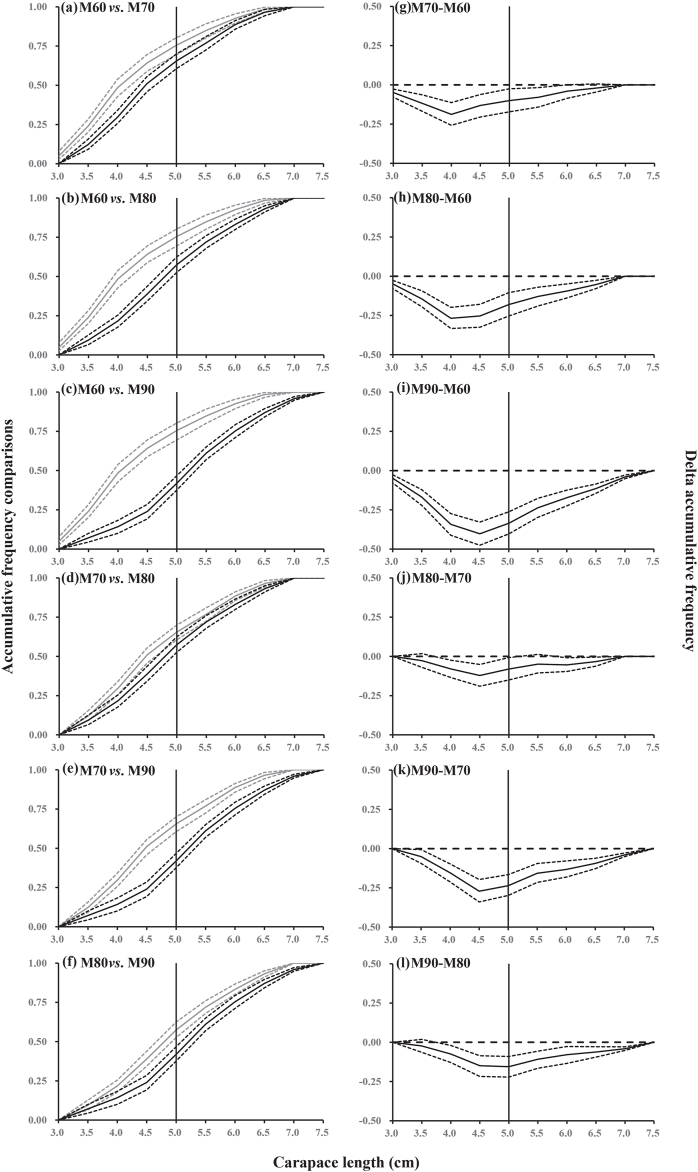
Cumulative length frequency distributions between the gillnets with four mesh sizes (60, 70, 80 and 90 mm). Left panel (a–f): cumulative length frequency distribution curves (solid lines) with 95 % confidence intervals (dotted lines) for the smaller gillnet mesh size (gray), and the larger mesh size (black). Right panel (g–l): cumulative length frequency distribution curves (solid lines) with 95 % confidence intervals (dotted lines) representing the differences between different gillnet mesh sizes. Vertical solid lines represent the minimum landing size of Asian paddle crab (*Charybdis japonica*). Horizontal dashed lines indicate no difference in accumulative length frequency between the two mesh sizes.

Generally, in the six pairwise comparisons between gillnets with the four different mesh sizes, the larger mesh size showed significantly lower and higher length frequency for undersized and legal-sized crabs, respectively (Fig. 5). The comparisons of the cumulative length frequency distributions also reflected a similar pattern (Fig. 6).

### 3.5 Species dominance

**Fig. 7:**
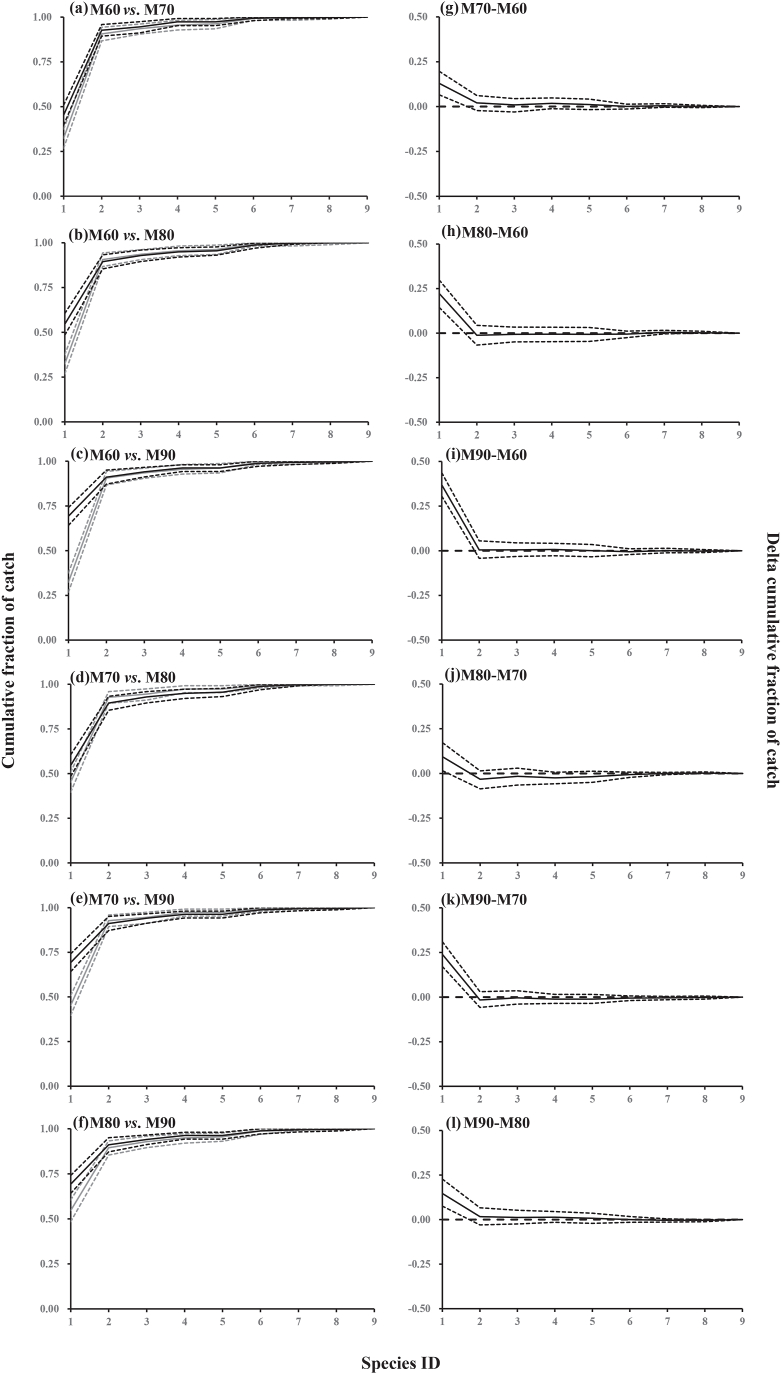
Cumulative species dominance curves for gillnets with the four mesh sizes (60, 70, 80 and 90 mm). Left panel (a–f): cumulative dominance curves (solid lines) with 95 % confidence intervals (dotted lines) for the species caught by the smaller gillnet mesh size (gray), and the larger mesh size (black). Right panel (g–l): pairwise difference (delta) for cumulative dominance curves (solid lines) with 95 % confidence intervals (dotted lines) representing the differences in the cumulative species dominance between different gillnet mesh sizes. Horizontal dashed lines are baseline for no significant difference in cumulative species dominance between the two mesh sizes. The x-axis shows the species ID: 1 Legal-sized *C. japonica*, 2 Undersized *C. japonica*, 3 *S. schlegelii*, 4 *H. otakii*, 5 *M*. *nudus*, 6 *H. agrammus*, 7 *P. yokohamae*, 8 *P*. *pectinifera*, 9 *P. bicoloratus*.

*C. japonica* was the most dominant species captured by gillnets of all mesh sizes (Table 5; Fig. 7). Specifically, the total percentage of undersized and legal-sized *C. japonica* exceeded 90 % in gillnets of all mesh sizes, while the contribution of all bycatch species to the catch composition was below 10 % (Table 5). However, during the experiments, seven bycatch species were observed in the gillnet catches. No significant differences in catch composition were observed for the bycatch species in this study when comparing gillnets of the four different mesh sizes (Table 5; Fig. 7).

**Table 5:** Species dominance values (in %) for the gillnets with four mesh sizes (95 % confidence intervals in brackets).

Species	M60	M70	M80	M90
Legal-sized *Charybdis japonica*	32.44 (26.67–37.56)	45.34 (40.24–51.05)	54.66 (48.70–60.15)	69.29 (63.98–74.51)
Undersized *Charybdis japonica*	58.22 (52.67–64.10)	47.37 (41.55–52.69)	34.82 (30.04–39.29)	21.85 (17.28–26.22)
*Sebastes schlegelii*	2.89 (0.97–5.22)	1.82 (0.42–3.58)	3.44 (1.31–5.96)	2.95 (1.05–5.15)
*Hexagrammos otakii*	2.00 (0.46–4.02)	2.83 (1.00–4.93)	2.02 (0.62–3.93)	2.17 (0.40–4.39)
*Mesocentrotus nudus*	0.67 (0.00–1.73)	0.00 (0.00–0.00)	0.61 (0.00–1.72)	0.00 (0.00–0.00)
*Hexagrammos agrammus*	3.11 (0.98–5.59)	2.02 (0.60–4.01)	3.24 (1.26–5.66)	2.56 (1.14–4.40)
*Pseudopleuronectes yokohamae*	0.00 (0.00–0.00)	0.40 (0.00–1.38)	1.01 (0.00–2.55)	0.59 (0.00–1.56)
*Patiria pectinifera*	0.44 (0.00–1.43)	0.00 (0.00–0.00)	0.20 (0.00–1.00)	0.20 (0.00–0.97)
*Platichthys bicoloratus*	0.22 (0.00–0.98)	0.20 (0.00–1.00)	0.00 (0.00–0.00)	0.39 (0.00–1.30)

Fig. 7 shows dominance curves for the cumulative dominance values for gillnets with different mesh sizes. The horizontal parts of the cumulative dominance curves (Fig. 7) showed that the bycatch species were less represented in the catch composition in all gillnets while the steep parts of the cumulative dominance curve for the first two ranked species showed dominance by legal-sized and undersized *C. japonica*, respectively (Fig. 7). Significant differences in catch composition were observed for undersized and legal-sized *C. japonica* when comparing the gillnets with different mesh sizes. Specifically, the pairwise difference in cumulative catch dominance curves showed the dissimilarity between the catch composition of different mesh sizes with less undersized *C. japonica* dominating the catches of gillnets with larger mesh sizes while the opposite was observed for the legal-sized crabs (Fig. 7). These results were in line with the results described above regarding the *C. japonica* capture probability for gillnets with different mesh sizes.

## 4 Discussion

To the best of our knowledge, this is the first study estimating the capture probability and catch composition in *C. japonica* gillnet fishery using different gillnet mesh sizes. Since the optimal gillnet mesh size for this fishery is not yet scientifically established and due to the bycatch and discard issues of undersized crabs, the results of this study can serve as technical guidelines for improving sustainable exploitation of *C. japonica* in the gillnet fishery.

Our results demonstrated that increasing the gillnet mesh size in this fishery could greatly improve the capture of legal-sized *C. japonica* while reducing capture of undersized individuals as reflected by the length-dependent capture probability curves, exploitation pattern indicators, and length frequency distributions. For instance, compared with the M60, gillnets with larger mesh sizes showed lower capture probability for undersized crabs and higher capture probability for legal-sized individuals. Furthermore, this was also reflected by lower *NP*- and *ndRatio* values and higher *NP* + values, and right shifted length frequency distributions, showing that gillnets with larger mesh sizes captured larger number of legal-sized crabs. These findings were in line with the earlier studies in other gillnet fisheries targeting different crab species. Specifically, Park et al. [21] and Xu et al. [14] reported that increasing mesh sizes could improve the gillnet selectivity for swimming crab (*Portunus trituberculatus*).

The improved capture probability of legal-sized crabs by gillnets with larger mesh sizes can be caused by the following reasons. First, although the crabs are more prone to be caught by entangling in gillnets than fish species [14], this capture mode probability for crabs may also be dependent on the matching degree between crabs’ morphological characteristics and gillnet design parameters (e.g., mesh openings, twine diameter, and hanging ratio), including the mesh size [34,41]. Secondly, this size dependency can be explained by the behavioral and physiological characteristics of *C. japonica* of different life stages. Generally, adult crustaceans often possess a more developed visual system than juvenile individuals to enable conducting more elaborate tasks, such as navigation and spatial vision [42]. Therefore, under specific fishing environments, small mesh size may be more easily perceived and detected by adult individuals who then would avoid contacting the gear. Further, the undersized crabs may become easier enmeshed in a smaller mesh size netting compared to legal-sized crabs. Therefore, gillnet sheets with small mesh size can have greater capture probability of small individuals.

The MMS regulation is one of China's most important input controls to protect fishery resources [43]. These regulations should be based on the selective properties of fishing gear to improve the capture of legal-sized individuals while at the same time reducing the bycatch of undersized conspecifics. Based on the results observed in this study using gillnets of 60, 70, 80 and 90 mm mesh size, which is the usual mesh size range in this fishery, we recommend 90 mm as the optimal MMS for capturing *C. japonica* in gillnet fisheries in the Yellow Sea of China. Specifically, for M90 gillnets, the exploitation pattern indicator (*ndRatio*_*m*_) showed that the proportion of undersized crabs in the catches was 23.97 % (CI: 19.26–28.51 %). This is in accordance with the current bycatch ratio regulation for this fishery and, therefore, would be favorable for sustainable use of this resource. Furthermore, lower capture probability for undersized crabs would improve the operational efficiency for the fishers by reducing the associated work of removing undersized crabs from the gillnets and avoiding gillnet damages. Last but not least, the M90 gillnets showed significantly higher catch efficiency for legal-sized crabs compared to the smaller mesh size nets which could increase the income in this fishery due to high market prices of large compared to smaller crabs. It should be noted that in this study we testes the mesh size range that is commonly used in the commercial fisheries targeting *C. japonica*. Of these mesh sizes, M90 was selected as the optimal configuration. However, using even larger mesh sizes than M90 may further improve the fishing performance in this fishery. Therefore, further investigations are encouraged to evaluate the impact of a wider range of mesh sizes on this fishery.

It is noteworthy that mesh size has been identified as an important factor affecting the catch composition, including the bycatch proportion, in gillnet fisheries [22,23]. Using large mesh sizes can potentially capture large non-targeted marine animals including threatened species, while using small mesh sizes may result in the capture of juvenile target- and small bycatch species [44]. It is, therefore, essential to assess the catch composition of different mesh sizes for various fish species that are abundant in the fishery. In this study, we found that altering the gillnet mesh size did not show any significant differences in catch composition regarding the bycatch species observed. Therefore, changing the gillnet mesh size did not increase the risk of capturing bycatch species. Furthermore, the dominance of the bycatch species observed in this study was relatively low compared to *C. japonica*. In this fishery, the interspecific interactions may have a significant impact on the catch composition observed. Because of the strong territorial and aggressive behavior of *C. japonica*, the high density of crabs in the adjacent area of gillnets may prevent the approaching bycatch species [7,45].

Some precautions are needed regarding the results obtained in this study since they are based on ten deployments which leads to uncertainty in the estimated length-dependent capture probability curves, exploitation pattern indicators, length frequency distributions, and species dominance results. However, sample size of ten deployments is not unusual in published scientific literature for gear selectivity and fishing efficiency studies [[46], [47], [48], [49], [50], [51], [52]], and the uncertainties are reflected in the confidence intervals. Therefore, it is feasible to make conclusions based on the results obtained in this study as long as the confidence intervals are considered.

In this study, we estimated the effect of changing the gillnet mesh size in the *C. japonica* fishery, keeping other technical parameters such as hanging ratio, twine thickness, net material, filament type, and twine color [18] similar. However, these other gillnet technical parameters, in addition to mesh size, are significant factors affecting the capture modes and selective properties of gillnets that should further be examined to improve the sustainability in the *C. japonica* fishery. Previous research has mainly explored the effects of these factors on the selectivity and capture efficiency of various fish species at local and regional scales in different fishing areas [53]. Considering the important role of crab gillnet fisheries along the coast of China as well as other regions worldwide, more sea trials are needed to focus on these factors and their interactions in future work to fill the knowledge gaps in these fisheries.

## Funding statement

This study was supported by the Project of Marine and Fishery Technology Innovation of Shandong (No. 2017HYCX007).

## Data availability statement

Data will be made available on request.

## CRediT authorship contribution statement

**Mengjie Yu:** Writing – review & editing, Writing – original draft, Visualization, Validation, Resources, Methodology, Investigation, Formal analysis, Data curation, Conceptualization. **Bent Herrmann:** Writing – review & editing, Writing – original draft. **Kristine Cerbule:** Writing – review & editing, Writing – original draft, Visualization, Validation, Resources, Methodology, Formal analysis, Data curation. **Changdong Liu:** Writing – original draft, Validation, Supervision, Resources, Conceptualization. **Liyou Zhang:** Resources, Investigation. **Yanli Tang:** Writing – original draft, Validation, Supervision, Resources, Project administration, Funding acquisition, Conceptualization.

## Declaration of competing interest

The authors declare that they have no known competing financial interests or personal relationships that could have appeared to influence the work reported in this paper.
